# Effect of sensorimotor orthoses on rearfoot motion in patients with Charcot-Marie-Tooth disease: a pilot study

**DOI:** 10.1186/1757-1146-7-S1-A88

**Published:** 2014-04-08

**Authors:** Caleb Wegener, Katrin Wegener, Karl-Heinz Schott, Joshua Burns

**Affiliations:** 1 Discipline of Exercise and Sports Science, Faculty of Health Sciences, The University of Sydney, NSW, 1825, Australia; 2 Shoe Tech Pty. Ltd, Pedorthic Clinic, Dee Why, NSW, 2099, Australia; 3 Faculty of Health Sciences, The University of Sydney/ Institute for Neuroscience and Muscle Research, The Children’s Hospital at Westmead, Sydney, NSW, 2145, Australia

## Background

Charcot-Marie-Tooth disease (CMT) is the most common hereditary peripheral neuropathy, with an incidence of 1 in 2,500 [1]. CMT is characterised by the progressive weakening of the distal muscles and sensory loss of the limbs, particularly around the foot and ankle resulting in balance, walking impairments, cavus foot deformity and lateral instability [2,3]. Clinical anecdotes suggest foot orthoses designed on the ‘sensorimotor’ paradigm proposed by Lothar Jahrling are beneficial at improving lateral stability during gait in patients with CMT. The purpose of this study was to investigate the effect of sensorimotor orthoses on frontal plane ankle motion in people with CMT.

## Methods

Four males and one female with CMT aged 31 to 64 years volunteered for the study. Each participant were fitted with an extra depth prefabricated pedorthic shoe (Gadean Walker Stretch, Malaga, WA, Australia) and a custom made orthoses prescribed according to the sensorimotor paradigm. Participants completed five walking trials at a self-selected velocity while wearing the shoe and shoe with orthoses in a randomised order. Three-dimensional ankle joint complex motion was measured using a motion-analysis system. Rearfoot motion was attained by detachable wand triad-marker through a window in the heel counter of the shoe. Data were time-normalised by linear interpolation to the stance phase and ensemble-averaged across trials and participants. Maximum and mean frontal plane motion from initial contact until 50% of stance was calculated. Paired sample t-tests were undertaken to assess significance between conditions. Participants were asked to nominate which condition felt more stable during walking.

## Results

Gait velocity was not altered between the shoe (1.16m/s (0.13)) and orthoses (1.7m/s (0.12), *p*=0.537). Mean ankle eversion increased during loading while wearing orthoses (mean change 3.7° (2.8), *p*=0.041). Maximum ankle eversion increased during loading while wearing orthoses (mean change 3.6° (2.9), *p*=0.051). All five participants reported a sense of increased stability while walking with the orthoses.

## Conclusions

Sensorimotor orthoses increase ankle eversion in people with CMT and may provide increased gait stability during the loading phase of gait.
